# Non-linear Association Between Body Mass Index and Ventricular Tachycardia/Ventricular Fibrillation in Patients With an Implantable Cardioverter-Defibrillator or Cardiac Resynchronization Therapy Defibrillator: A Multicenter Cohort Study

**DOI:** 10.3389/fcvm.2020.610629

**Published:** 2020-11-30

**Authors:** Bin Zhou, Shuang Zhao, Min Tang, Keping Chen, Wei Hua, Yangang Su, Jiefu Yang, Zhaoguang Liang, Wei Xu, Shu Zhang

**Affiliations:** ^1^Arrhythmia Centre, Fuwai Hospital, National Centre for Cardiovascular Diseases, National Clinical Research Center of Cardiovascular Diseases, State Key Laboratory of Cardiovascular Disease, Chinese Academy of Medical Sciences and Peking Union Medical College, Beijing, China; ^2^Department of Cardiology, Shanghai Institute of Cardiovascular Diseases, Zhongshan Hospital, Fudan University, Shanghai, China; ^3^Department of Cardiology, Beijing Hospital, Beijing, China; ^4^Department of Cardiology, First Affiliated Hospital of Harbin Medical University, Harbin, China; ^5^Department of Cardiology, Nanjing Drum Tower Hospital, Nanjing, China

**Keywords:** body mass index, sudden cardiac death, ventricular tachycardia, implantable cardioverter-defibrillator, non-linearity

## Abstract

**Background:** Results from studies on the effects of obesity on sudden cardiac death (SCD) or ventricular tachycardia/ventricular fibrillation (VT/VF) in patients with an implantable cardioverter-defibrillator/cardiac resynchronization therapy defibrillator (ICD/CRT-D) are inconsistent. Our study aimed to explore the impact of BMI on VT/VF in patients with an ICD/CRT-D.

**Methods:** We retrospectively analyzed the data from the Study of Home Monitoring System Safety and Efficacy in Cardiac Implantable Electronic Device–implanted Patients in China. Nine hundred and seventy ICD/CRT-D patients were enrolled. The outcome was the first occurrence of VT/VF requiring appropriate ICD/CRT-D therapy. A general linear model and general additive model were used to assess the relationship between BMI and VT/VF.

**Results:** After a median follow-up of 5.17 years, 352 (36.3%) patients experienced VT/VF requiring appropriate ICD/CRT-D therapy. BMI, whether as a continuous variable or a categorical variable classified by various BMI classification criteria, had no significant effect on VT/VF according to a multivariable Cox proportional hazards model with adjustment for potential confounders. However, a non-linear association between BMI and VT/VF was identified using a cubic spline function model and smooth curve fitting. The inflection point for the curve was found at a BMI level of 23 kg/m^2^. The hazard ratios (95% confidence intervals) for VT/VF were 1.12 (1.01–1.24) and 0.96 (0.90–1.02) to the left and right of the inflection point, respectively.

**Conclusions:** BMI is related to VT/VF in a non-linear manner in patients with an ICD/CRT-D. Our research suggests a complicated role of BMI in VT/VF with different impacts at different ranges.

## Introduction

Sudden cardiac death (SCD) is a global public health concern, accounting for up to 50% of all cardiovascular deaths (1). The exact definition of SCD is sudden and unexpected death occurring within an hour of the onset of symptoms or occurring in patients found dead within 24 h of being asymptomatic presumably due to a cardiac arrhythmia or hemodynamic catastrophe (2). Fatal ventricular tachycardia/ventricular fibrillation (VT/VF) plays vital roles in the development of SCD and results in hemodynamic collapse with cessation of cardiac mechanical activity (2). Current clinical practice guidelines recommend the implantation of an implantable cardioverter-defibrillator/cardiac resynchronization therapy defibrillator (ICD/CRT-D) to treat possible ventricular VT/VF in the management of patients at high risk of SCD (2–4). For patients with an ICD/CRT-D, VT/VF requiring appropriate ICD/CRT-D therapy is a commonly used surrogate for SCD (5, 6).

Additionally, as a global health problem, obesity, which is usually assessed by body mass index (BMI) in clinical practice, has been recognized as a risk factor for SCD in the general population (7). However, the results from the few studies on the effects of obesity on SCD or VT/VF in patients who had received an ICD/CRT-D due to their high risk of SCD have been controversial (6, 8–10).

Fully understanding the effect of obesity on the occurrence of SCD or VT/VF in patients with an ICD/CRT-D is conducive to risk stratification and helpful for guiding proper treatment for these patients. Our study intended to explore the impact of BMI on VT/VF requiring appropriate ICD/CRT-D therapy in patients with an ICD/CRT-D.

## Methods

### Study Design, Setting, and Population

**Figure 1:**
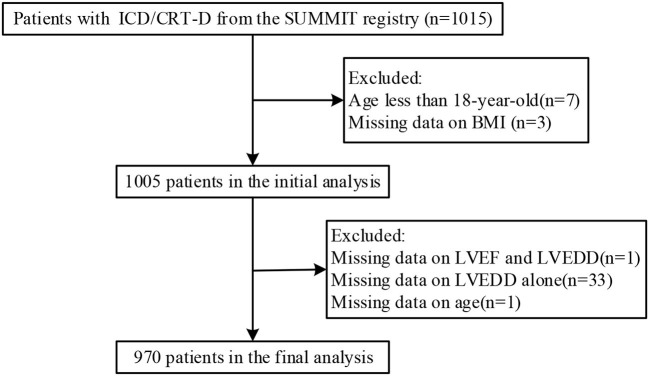
Flowchart of the study population. Abbreviations are shown in Table 1.

Study of Home Monitoring System Safety and Efficacy in Cardiac Implantable Electronic Device–implanted Patients (SUMMIT) was a prospective, observational, multicenter registry used to evaluate the safety and efficacy of a cardiac implantable electronic device with a home monitoring (HM) system in China. We performed a retrospective cohort study based on data from the SUMMIT registry. A total of 1,015 patients who underwent ICD or CRT-D implantation with an HM system (Biotronik, Berlin, Germany) between May 2010 and May 2015 from the SUMMIT registry were included. Next, we excluded patients meeting any of the following criteria: (1) patients younger than 18 years (*n* = 7); (2) patients with missing body mass index (BMI) data (*n* = 3); (3) patient with missing data on left ventricular ejection fraction (LVEF) and left ventricular end-systolic dimension (LVEDD) (*n* = 1); (4) patients with missing data on LVEDD alone (*n* = 33); and (5) patient with missing data on age (*n* = 1). Thus, 970 patients were enrolled in the final analysis. The flowchart of the study population is shown in Figure 1. According to the clinical practice guidelines (2–4), all patients were satisfied with indications of primary prevention or secondary prevention of SCD. Primary prevention of SCD refers to the use of ICDs in individuals who are at risk for but have not yet had an episode of sustained VT, VF, or resuscitated cardiac arrest; secondary prevention refers to the prevention of SCD in patients who have survived a prior sudden cardiac arrest or sustained VT or VF (3). A total of 394 patients satisfied the secondary prevention of SCD in our study. Among these patients, 98 (25%) had documented VF and resuscitated SCD, 236 (60%) had a history of documented sustained VT and 60 (15%) had a history of unexplained syncope and could be induced to VT or VF during electrophysiological study. The study protocols were approved by Ethics Committee of Fuwai Hospital, Chinese Academy of Medical Sciences (the chief institute) and all other participating organizations (Zhongshan Hospital, Fudan University et al.), and were in accordance with the Declaration of Helsinki. All patients signed informed consent forms before the study. All reporting followed the Strengthening the Reporting of Observational Studies in Epidemiology (STROBE) guidelines (11).

### Clinical Data Collection

BMI was calculated as weight (kg) divided by the square of the patient's height (m^2^) and shown as kg/m^2^. Because the BMI cutoff points for overweight and obesity vary under World Health Organization (WHO) criteria (12), Asian criteria (13), or Chinese criteria (14), and/or different BMI cutoff points were used in previous studies, we divided the BMI values into tertiles, which is a common and convenient method (15). Other baseline clinical characteristics, including age at implantation, gender, systolic blood pressure, diastolic blood pressure, indication of primary or secondary prevention, New York Heart Association (NYHA) class, implantation of ICD or CRT-D, ischemic cardiomyopathy, dilated cardiomyopathy, hypertrophic cardiomyopathy, Long QT syndrome, hypertension, diabetes, stroke, atrial fibrillation (AF), preimplant syncope, LVEF, LVEDD, β-blockers, amiodarone, angiotensin-converting enzyme inhibitor or angiotensin receptor blocker, loop diuretic, and aldosterone antagonists were acquired from the patients' medical records before ICD/CRT-D implantation. LVEF was calculated by using the modified Simpson's biplane rule.

### Device Settings and Outcome

The protocol of device programming settings was consistent with those of our previous study (16). The detailed protocol of the device programming settings is shown in Supplementary Material 1. The outcome was the first occurrence of VT/VF requiring appropriate ICD/CRT-D therapy based on data automatically transmitted to the HM system and as confirmed by two or more cardiologists through reviewing the intracardiac electrograms. Inappropriate events, VT with a heart rate slower than 140 bpm and non-sustained VT were excluded. Routine follow-ups were conducted, and patient status was confirmed via phone calls in the event that the transmission of their data was disrupted. The HM system recorded the interval from ICD/CRT-D implantation to the first occurrence of VT/VF requiring appropriate ICD/CRT-D therapy. The last time we assessed the VT/VF events in the HM system was June 2018.

### Statistical Analysis and Sensitivity Analysis

Data are presented as the means ± standard deviation or proportions. Chi-square test (categorical variables) or one-way analysis of variance with Bonferroni *post-hoc* test (continuous variables) was used to calculate differences between different BMI groups (tertiles). To investigate the association between BMI and VT/VF requiring appropriate ICD/CRT-D therapy, our statistical analyses consisted of 4 main steps.

Step 1: We plotted Kaplan-Meier curves to compare the outcomes of different BMI groups (log-rank test). Step 2: We used a generalized linear model such as the standard Cox proportional hazards model to assess the association between BMI and VT/VF. We constructed 4 Cox proportional hazards models: model 1, adjusted for none; model 2, adjusted for age and gender; model 3, adjusted for variables in model 2 plus variables that had a statistically significant effect on VT/VF at the 0.05 level in the univariate Cox model; and model 4, adjusted for all covariates presented in Table 1. Step 3: To address the non-linearity of the relation between BMI and an outcome, a generalized additive model was used. We conducted a cubic spline function model and smooth curve fitting (penalized spline method) to further explore the association between BMI and VT/VF. If non-linearity was detected, we first calculated the inflection point using a recursive algorithm and then constructed a 2-piecewise Cox proportional hazards model on both sides of the inflection point. We determined the best fit model (1-line Cox proportional hazards model vs. piecewise Cox proportional hazards model) based on the *P*-values for the log likelihood ratio test. Step 4: The subgroup analyses were performed using the Cox proportional hazards model. For continuous variables, we first converted them to categorical variables according to the clinical cutoff point and then performed an interaction test. Tests for effect modification by subgroup were based on interaction terms between subgroup indicators followed by likelihood ratio test.

**Table 1:** Baseline characteristics of study population according to BMI.

**Characteristics**	**Total (*n* = 970)**	**Tertile of BMI**			***P*-value**
		**Tertile 1 (<22.2 kg/m^**2**^) (*n* = 317)**	**Tertile 2 (22.2–24.4 kg/m^**2**^) (*n* = 312)**	**Tertile 3 (>24.4 kg/m^**2**^) (*n* = 341)**	
Age at implantation, years	60.3 ± 13.5	60.4 ± 14.6	60.3 ± 12.6	60.3 ± 13.4	0.988
Male	707 (72.9%)	198 (62.5%)	241 (77.2%)	268 (78.6%)	<0.001
SBP, mmHg	124.5 ± 17.4	124.0 ± 17.8	123.0 ± 16.5	126.4 ± 17.7	0.151
DBP, mmHg	76.9 ± 10.9	75.6 ± 11.3	76.5 ± 9.9	78.4 ± 11.2	0.002
Primary prevention	576 (59.4%)	187 (59.0%)	188 (60.3%)	201 (58.9%)	0.930
NYHA, class III/IV	484 (49.9%)	172 (54.3%)	146 (46.8%)	166 (48.7%)	0.148
CRT-D	266 (27.4%)	89 (28.1%)	91 (29.2%)	86 (25.2%)	0.503
Ischemic cardiomyopathy	324 (33.4%)	96 (30.3%)	98 (31.4%)	130 (38.1%)	0.069
Dilated cardiomyopathy	238 (24.5%)	83 (26.2%)	73 (23.4%)	82 (24.0%)	0.695
Hypertrophic cardiomyopathy	37 (3.8%)	9 (2.8%)	12 (3.8%)	16 (4.7%)	0.463
Long QT syndrome	12 (1.2%)	5 (1.6%)	3 (1.0%)	4 (1.2%)	0.777
Hypertension	305 (31.4%)	89 (28.1%)	92 (29.5%)	124 (36.4%)	0.049
Diabetes mellitus	101 (10.4%)	24 (7.6%)	34 (10.9%)	43 (12.6%)	0.101
Stroke	18 (1.9%)	3 (1.0%)	4 (1.3%)	11 (3.2%)	0.076
Atrial fibrillation	104 (10.7%)	38 (12.0%)	33 (10.6%)	33 (9.7%)	0.629
Pre-implant syncope	194 (20.0%)	67 (21.1%)	60 (19.2%)	67 (19.7%)	0.820
LVEF, %	42.5 ± 14.9	41.6 ± 15.0	42.9 ± 15.0	42.8 ± 14.8	0.476
LVEDD, mm	58.8 ± 13.1	58.1 ± 12.8	58.8 ± 13.5	59.6 ± 13.0	0.301
β-Blocker	566 (58.4%)	177 (55.8%)	181 (58.0%)	208 (61.0%)	0.402
Amiodarone	290 (29.9%)	91 (28.7%)	104 (33.3%)	95 (27.9%)	0.266
ACEI or ARB	360 (37.1%)	128 (40.4%)	100 (32.1%)	132 (38.7%)	0.073
Loop diuretic	280 (28.9%)	84 (26.5%)	93 (29.8%)	103 (30.2%)	0.523
Aldosterone antagonists	363 (37.4%)	125 (39.4%)	105 (33.7%)	133 (39.0%)	0.246

*Continuous data and categorical data were given as mean ± SD and number (percentage), respectively*.

*ACEI, angiotensin-converting enzyme inhibitor; ARB, angiotensin receptor blocker; BMI, body mass index; CRT-D, cardiac resynchronization therapy defibrillator; DBP, diastolic blood pressure; LVEF, left ventricular ejection fraction; LVEDD, left ventricular end-systolic dimension; NYHA, New York Heart Association; SBP, systolic blood pressure*.

To ensure the robustness of the data analysis, we performed the following sensitivity analysis. (1) We compared the complete dataset and missing dataset, and the results demonstrated that nearly all variables were similar, showing that the selection bias was relatively small (Supplementary Table 1). (2) We converted the BMI into a categorical variable by tertiles and calculated the *P* for trend. The purpose was to verify the results of BMI as a continuous variable and to observe the possibility of non-linearity. (3) We performed the same analysis in steps 1 and 2 using the BMI classification based on WHO criteria, Asian criteria or Chinese criteria.

All analyses were performed using R version 4.0.0 (R Foundation for Statistical Computing, Vienna, Austria) and Empower (R) (X&Y Solutions, Inc., Boston, MA). All *P* < 0.05 (two-sided) were considered statistically significant.

## Results

### Baseline Characteristics of the Participants

After exclusions, the final analysis dataset consisted of 970 adults (mean age at implantation: 60.34 ± 13.50 years, 72.89% male). The distribution of the baseline characteristics of the study population according to BMI tertiles is shown in Table 1. The ranges of BMI for tertiles 1 through 3 were <22.2, 22.2–24.4, and >24 kg/m^2^, respectively. There were nearly no significant differences between BMI tertiles for all included characteristics except for gender, diastolic blood pressure and presence of hypertension. Compared with those in T1 and T2 of the BMI, the participants in T3 were more likely to be male, have a higher diastolic blood pressure and present with hypertension (all *p* < 0.05).

### Association Between BMI and VT/VF Using Kaplan-Meier Curves and the Cox Proportional Hazards Models

**Figure 2:**
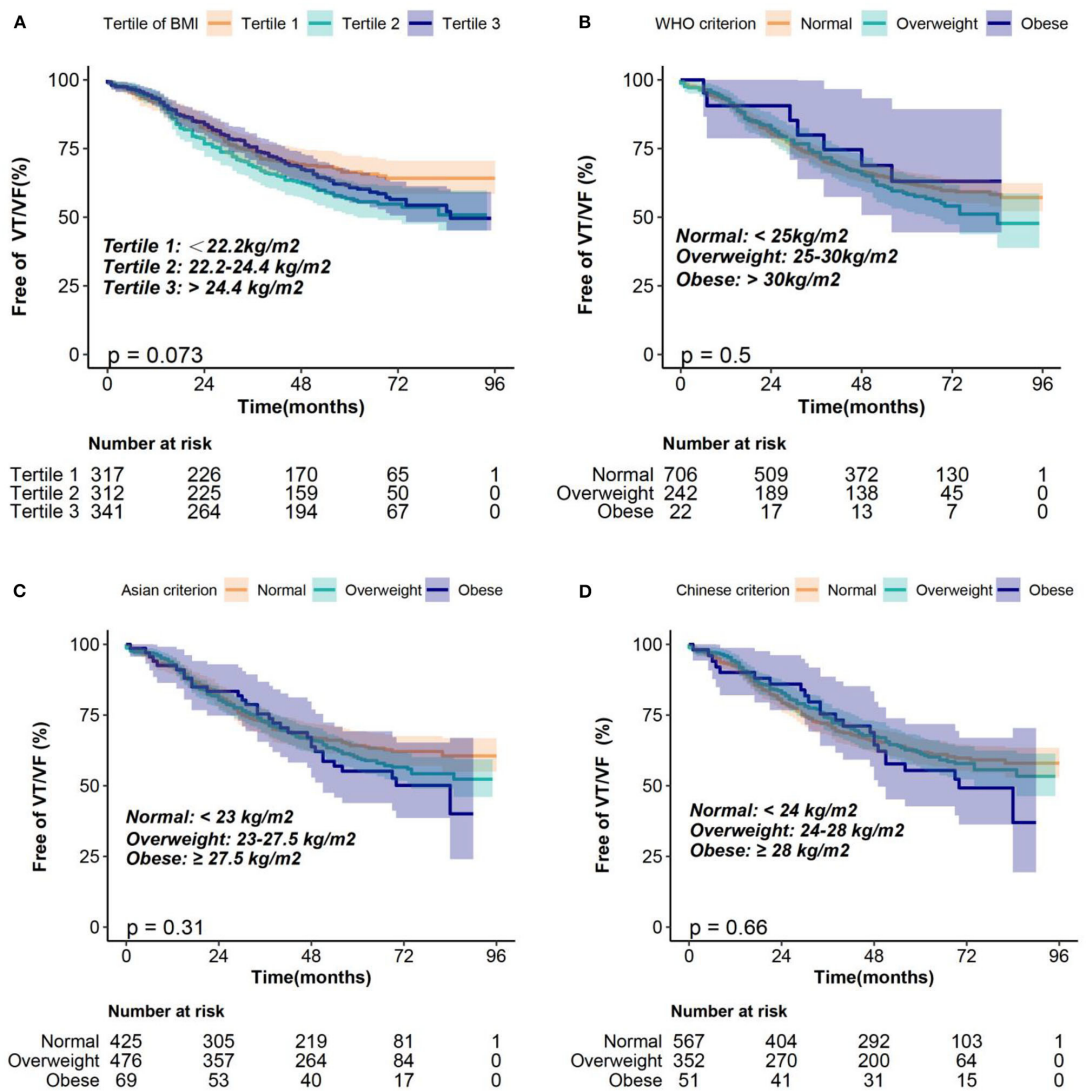
Kaplan–Meier estimates of the probability of being free from VT/VF according to the BMI classification of **(A)** tertiles, **(B)** WHO criterion, **(C)** Asian criterion, **(D)** Chinese criterion. BMI, body mass index; VT/VF, ventricular tachycardia/ventricular fibrillation; WHO, World Health Organization.

The median follow-up duration was 5.17 (interquartile, 4.3–5.83) years. During the follow-up, 352 (36.3%) patients experienced VT/VF requiring ICD/CRT-D therapy. Kaplan–Meier survival curves were plotted to determine the probability of patients being VT/VF free according to BMI tertile (Figure 2). The results showed that the probability of being free from VT/VF for patients in the BMI tertiles was not significantly different (log-rank, *p* = 0.073). Additionally, we plotted the Kaplan–Meier survival curves using different BMI classification criteria for clinical applications, and the results were consistent with those using BMI tertiles (Figure 2). Univariate Cox proportional hazards models of VT/VF are shown in Supplementary Table 2. Older age, male, AF, lower LVEF and wider LVEDD were associated with higher risk of VT/VF in the univariate Cox proportional hazards models (*P* < 0.05). Then, we constructed 4 Cox proportional hazards models to analyze the independent role of BMI in VT/VF. The HRs and 95% CIs for these 4 models are listed in Table 2.

**Table 2:** Association of BMI with VT/VF in different models.

**BMI (kg/m^**2**^)**		**Model 1**		**Model 2**		**Model 3**		**Model 4**	
	**No. of VT/VF**	**HR (95% CI)**	***P*-value**	**HR (95% CI)**	***P*-value**	**HR (95% CI)**	***P*-value**	**HR (95% CI)**	***P*-value**
**Continuous**	352	1.04 (1.00, 1.07)	0.0364	1.03 (0.99, 1.07)	0.1315	1.03 (0.99, 1.06)	0.1662	1.03 (0.99, 1.07)	0.1510
**Tertiles**									
<22.1	96	Reference		Reference		Reference		Reference	
22.1–24.4	126	1.36 (1.04, 1.77)	0.0233	1.28 (0.98, 1.67)	0.0702	1.29 (0.99, 1.69)	0.0616	1.30 (0.99, 1.71)	0.0565
>24.4	130	1.20 (0.92, 1.57)	0.1687	1.14 (0.87, 1.48)	0.3517	1.13 (0.87, 1.48)	0.3541	1.11 (0.84, 1.46)	0.4643
*P*_trend_-value			0.1899		0.3975		0.4072		0.5405
**WHO criterion**									
<25	245	Reference		Reference		Reference		Reference	
25–30	100	1.13 (0.90, 1.43)	0.3017	1.11 (0.88, 1.40)	0.3786	1.11 (0.88, 1.41)	0.3662	1.08 (0.85, 1.38)	0.5123
≥30	7	0.84 (0.40, 1.79)	0.6598	0.83 (0.39, 1.75)	0.6170	0.73 (0.34, 1.56)	0.4177	0.80 (0.36, 1.78)	0.2562
*P*_trend_-value			0.6169		0.7284		0.8864		0.8235
**Asian criterion**									
<23	138	Reference		Reference		Reference		Reference	
23–27.5	183	1.14 (0.92, 1.42)	0.2406	1.08 (0.87, 1.36)	0.4791	1.09 (0.87, 1.37)	0.4384	1.06 (0.84, 1.34)	0.6014
≥27.5	31	1.29 (0.88, 1.91)	0.1943	1.24 (0.84, 1.84)	0.2749	1.22 (0.83, 1.81)	0.3177	1.25 (0.83, 1.87)	0.2848
*P*_trend_-value			0.1280		0.2544		0.2691		0.3018
**Chinese criterion**									
<24	197	Reference		Reference		Reference		Reference	
24–28	131	1.01 (0.81, 1.26)	0.9188	0.98 (0.79, 1.22)	0.8613	0.97 (0.78, 1.21)	0.8057	0.94 (0.75, 1.18)	0.6017
≥28	24	1.22 (0.80, 1.86)	0.3657	1.17 (0.77, 1.80)	0.4565	1.13 (0.74, 1.73)	0.5802	1.20 (0.77, 1.88)	0.4246
*P*_trend_-value			0.5240		0.7202		0.8452		0.8012

*Model 1: adjusted for none. Model 2: adjusted for age, gender. Model 3: adjusted for variables in Model 2 plus atrial fibrillation, LVEF, LVEDD. Model 4 adjusted for all covariates presented in Table 1. CI, confidence interval; HR, hazard ratio; VT/VF, ventricular tachycardia /ventricular fibrillation; WHO, World Health Organization; other abbreviations are shown in Table 1*.

In the unadjusted model (model 1), each 1 kg/m^2^ BMI increase was associated with a 4% increased risk of VT/VF. However, in model 2, after adjusting for age and gender, the association between BMI and the risk of VT/VF was not statistically significant. Additionally, after adjusting for additional covariates in model 3 (adjusting for age, gender, AF, LVEF, and LVEDD) and all covariates presented in Table 1 in model 4, the results negligibly changed. We also converted BMI from a continuous variable to a categorical variable (tertiles). Compared with participants in T1 of the BMI, there was no significant increased risk of VT/VF for patients in either T2 or T3 in the adjusted models (models 2–4). The *P* trend value was not significant in any of the models, indicating a possible non-linear association between BMI and VT/VF. Moreover, we also performed sensitivity analyses using different BMI classification criteria for clinical applications, and the results were nearly the same as those based on the BMI tertiles.

### Association Between BMI and VT/VF Using the Cubic Spline Function Model and Smooth Curve Fitting

**Figure 3:**
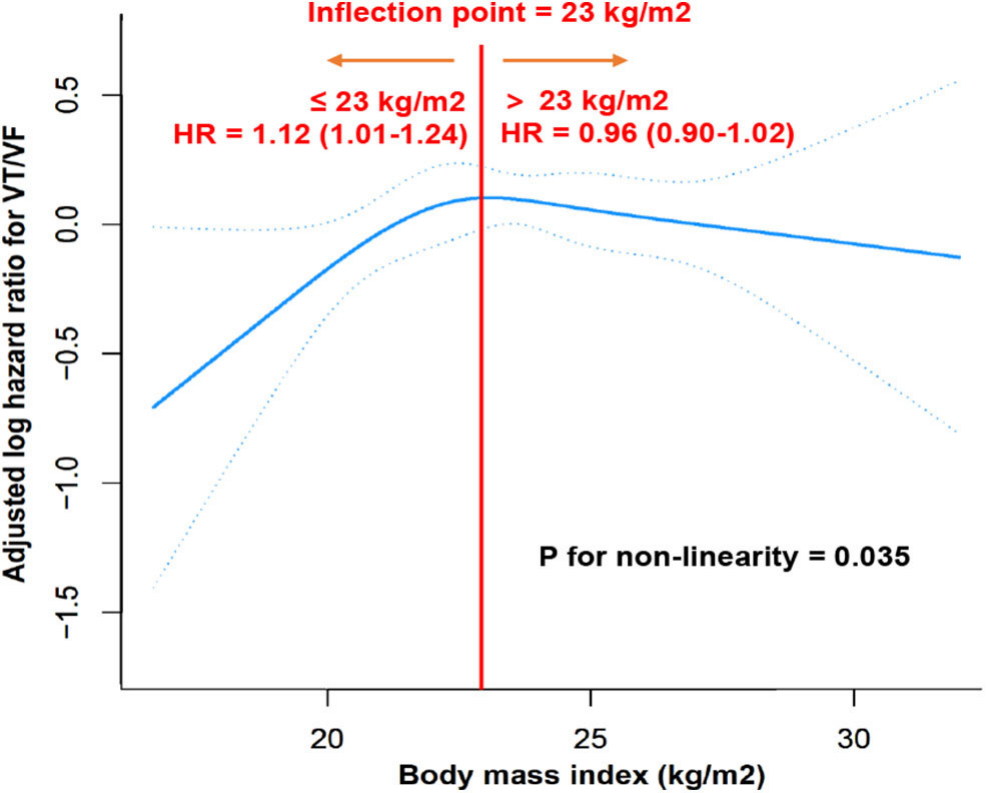
Dose response relationship of BMI and VT/VF. A non-linear association between BMI and VT/VF was found (*P* for non-linearity = 0.035) in a generalized additive model. The solid blue line and dashed blue line represent the estimated values and their corresponding 95% CI. Adjustment factors included all covariates presented in Table 1. The inflection point detected for BMI was 23 kg/m^2^. When BMI was ≤23 kg/m^2^, HR per unit (kg/m^2^) higher BMI was 1.12 (95% CI 1.01–1.24). However, When BMI was > 23 kg/m^2^, higher BMI did not add risk of VT/VF but showed a trend of decreased risk of VT/VF (HR 0.96, 95% CI 0.90–1.02). BMI, body mass index; CI, confidence interval; HR, hazard ratio; VT/VF, ventricular tachycardia/ventricular fibrillation.

We conducted a cubic spline function model and smooth curve fitting (penalized spline method) to visualize the relationship between BMI and VT/VF. The fully adjusted smooth curve fitting showed a non-linear association between BMI and VT/VF (Figure 3). We further conducted a threshold effect analysis of BMI on VT/VF. We fitted the association between BMI and VT/VF using a 1-line Cox proportional hazards model and a 2-piecewise Cox proportional hazards model, respectively. The *P-*value for the log likelihood ratio test was <0.05, indicating that the 2-piecewise Cox proportional hazards model was more suitable for fitting the association between BMI and VT/VF. As shown in Figure 3, a non-linear association between BMI and VT/VF was found (*P* for non-linearity = 0.035). The inflection point that we detected for the BMI was 23 kg/m^2^. When the BMI was ≤23 kg/m^2^, the hazard ratio (HR) per unit (kg/m^2^) of higher BMI was 1.12 [95% confidence interval (CI) 1.01–1.24]. However, when the BMI was >23 kg/m^2^, the higher BMI did not add to the risk of VT/VF but showed a trend of decreased risk of VT/VF (HR 0.96, 95% CI 0.90–1.02).

### Subgroup Analysis

**Figure 4:**
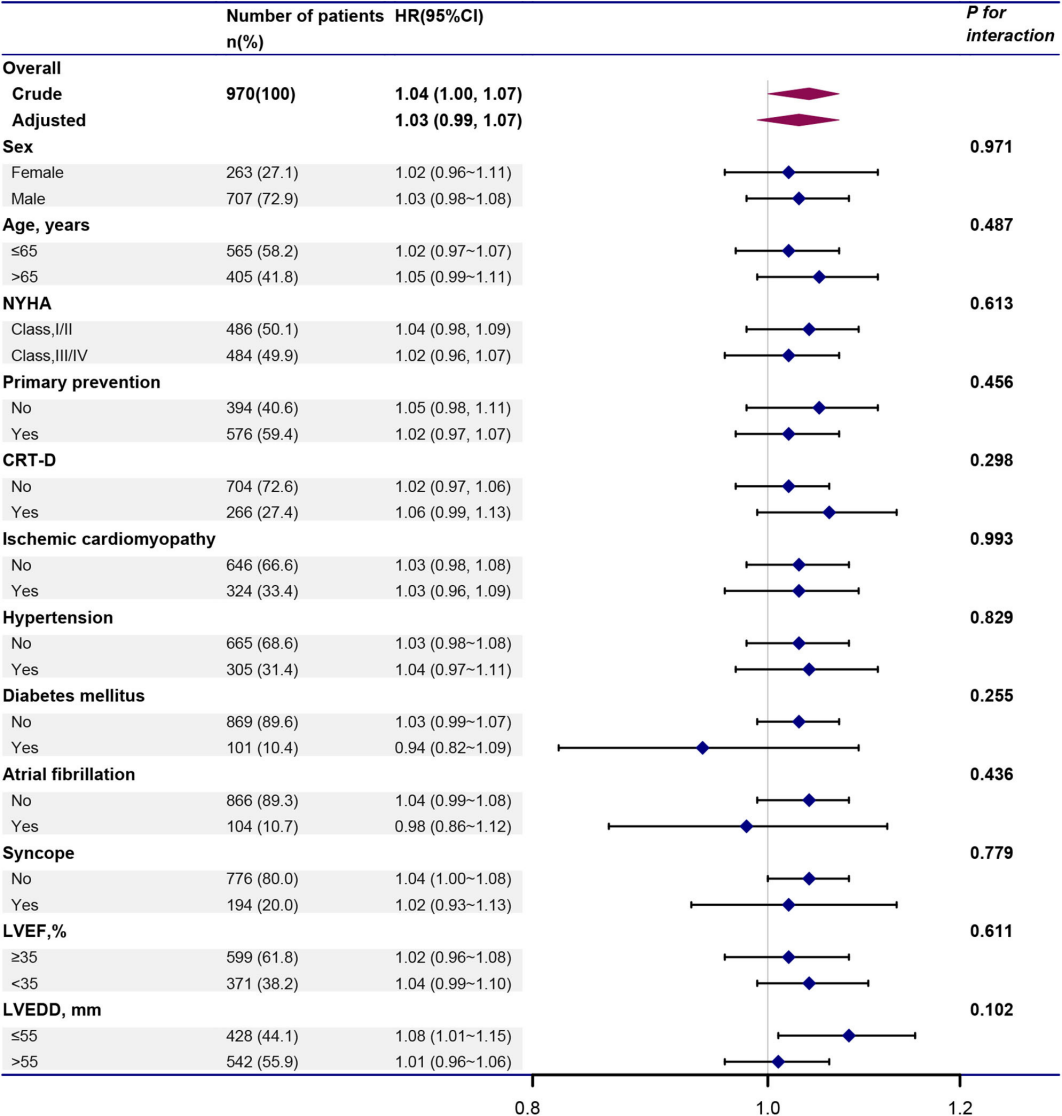
Forest plot illustrating the HR and 95% CI of BMI and VT/VF in total population and various subgroups. Above models adjusted for all covariates presented Table 1. In each subgroup, the model is not adjusted for the stratification variable. CI, confidence interval; HR, hazard ratio; VT/VF, ventricular tachycardia/ventricular fibrillation. Other abbreviations are shown in Table 1.

We further investigated the role of other covariates between BMI and VT/VF. As shown in Figure 4, the association between BMI and VT/VF was consistent in the following subgroups: sex, age, NYHA, primary prevention, CRT-D, ischemic cardiomyopathy, hypertension, diabetes mellitus, AF, syncope, LVEF and LVEDD (all *P*-values for these interactions >0.05).

## Discussion

The major findings of this study are as follows: (1) BMI, whether as a continuous variable or a categorical variable classified by various BMI classification criteria, had no significant impact on VT/VF in the ICD/CRT-D patients according to Kaplan-Meier curves and Cox proportional hazards models. (2) A non-linear association between BMI and VT/VF was identified using a cubic spline function model and smooth curve fitting. When the BMI was ≤23 kg/m^2^, a higher BMI was associated with a higher risk of VT/VF. When the BMI was >23 kg/m^2^, a higher BMI did not increase the risk of VT/VF but showed a trend of decreased risk of VT/VF.

Several studies have illustrated the effect of BMI on the risk of VT/VF in ICD/CRT-D patients, but the results have been inconsistent (6, 8–10). Pietrasik et al. conducted a retrospective analysis of non-diabetic patients with ischemic left ventricular dysfunction using data from the Multicenter Automatic Defibrillator Implantation Trial-II (MADIT II) and demonstrated that a higher rate of VT/VF was detected in obese patients (BMI ≥30 kg/m^2^) compared with the rate in non-obese patients (8). However, a *post-hoc* analysis of the ICD/CRT-D patients from the Multicenter Automatic Defibrillator Implantation trial with Cardiac Resynchronization Therapy (MADIT-CRT) showed that BMI had no impact on VT/VF (9). Another study from Spain also found that BMI was not associated with VT/VF in ICD patients for the purpose of primary prevention (10). These contradictory results may be the result of differences in the population characteristics, sample sizes, racial groups, and the adjustment of confounders. Moreover, these studies only investigated the linear relationship between BMI and VT/VF and did not address non-linear relationships. Gandhi et al. found an interesting phenomenon in their study that suggested an inverted U-shaped relationship between BMI and risk of VT/VF among ICD patients with systolic heart failure, with the highest risk found for the overweight BMI group (BMI 25–30 kg/m^2^) and the lower risk found in the normal group (BMI 18.5–25 kg/m^2^) and obese group (BMI ≥30 kg/m^2^) (6). However, this study did not investigate the non-linear relationship between BMI and VT/VF. The U-shaped relationship for BMI and VT/VF was based on the segmentation effect, which was not intuitive.

Our study used the Kaplan-Meier curve/Cox proportional hazards model or cubic spline function model/smooth curve fitting to explore the relationship between BMI and VT/VF. The results showed that BMI had a non-linear association with VT/VF. Had we used only a generalized linear model to identify the association between BMI and VT/VF, the way we classified the BMI would not have mattered: the result would be negative. Once the non-linear relationship is identified, it is not appropriate to use a generalized linear model to analyze the correlation. The cubic spline function model and smooth curve fitting were helpful in detecting the non-linear relationship between BMI and VT/VF. Additionally, the inflection point was identified to show the role of BMI in different intervals.

The mechanism resulting in this non-linear association is unclear. In the general population, obesity is associated with cardiac structural changes (i.e., cardiac hypertrophy and myocardial abnormalities such as fibrosis or fatty infiltration), electrical abnormalities (i.e., QT prolongation) and sleep apnea, which could lead to an increased risk of ventricular arrhythmias (17–20). However, the obesity paradox suggesting a positive association between a higher BMI and positive outcomes has been found in several cohorts, although the reason remains unclear (21–23). We suggest that there is a complicated interaction between the impact of obesity-related cardiac structural/electrical changes and obesity-related risk factors compared to the factors accounting for improved outcomes with obesity in patients at high risk of SCD. The non-linear association between BMI and VT/VF shows that the impact of BMI is quite different in different ranges. Perhaps when BMI is in a certain range (BMI ≤23 kg/m^2^ in our study), and the BMI is increased, then the harm caused by BMI dominates, which would lead to the increased risk of VT/VF; however, when BMI exceeds a certain range (BMI >23 kg/m^2^ in our study), the benefits conferred by the BMI play a leading role, and the risk of VT/VF exhibits a decreasing trend.

Our study has several strengths. First, our research is a multicenter study with a relatively large sample size and good generalizability. Second, we used both a generalized linear model and generalized additive model to explore the relationship between BMI and VT/VF to the maximum extent. Our results showed an interesting non-linear relationship between BMI and VT/VF. Third, we performed sensitivity analyses to enhance the robustness of the results. However, there are some limitations to our study. First, our study was an observational study that was subject to selection bias because patients without complete data were excluded. However, we sincerely compared the complete dataset and the missing dataset, and the results demonstrated that nearly all variables were similar, showing that the selection bias was relatively small. Second, our study did not collect data on obstructive sleep apnea and laboratory parameters which may have influence on the effect of BMI on VT/VF. We could not adjust for these substantial confounders; therefore, a prospective study collecting more variables may be necessary to validate our results. Third, the number of obese patients in our study was relatively small, which limited the generalizability of our results. Finally, we only had baseline BMI information, and data on changes in BMI were not collected during the follow-up. Higher fluctuations in BMI were related to adverse outcomes in patients with coronary heart disease (24). In the future, we will perform a prospective study enrolling a larger sample size to ensure the sample balance of each group and collect the change in BMI to better illustrate the influence of BMI at the baseline and upon dynamic changes in BMI on VT/VF in patients with an ICD/CRT-D.

## Conclusions

Our study identifies a non-linear relationship between BMI and VT/VF in patients with an ICD/CRT-D. Our research suggests a complicated role of BMI in VT/VF with different impacts at different ranges. Prospective studies are needed to better illustrate the role of BMI in VT/VF as well as the potential mechanisms accounting for this role.

## Data Availability Statement

The raw data supporting the conclusions of this article will be made available by the authors, without undue reservation.

## Ethics Statement

The study protocols were approved by ethics committee of Fuwai Hospital, Chinese Academy of Medical Sciences (the chief institute), and all other participating organizations (Zhongshan Hospital, Fudan University et al.), and were in accordance with the Declaration of Helsinki. All patients signed informed consent forms before the study. The patients/participants provided their written informed consent to participate in this study.

## Author Contributions

SZhang conceived and designed the research. MT, KC, WH, YS, JY, ZL, and WX conducted the ICD/CRT-D implantation. SZhao collected the data. BZ analyzed the data and wrote the manuscript. SZhang revised the manuscript. All authors read and approved the final manuscript.

## Conflict of Interest

The authors declare that the research was conducted in the absence of any commercial or financial relationships that could be construed as a potential conflict of interest.
