# Supplementary material for: Non-linear Association Between Body Mass Index and Ventricular Tachycardia/Ventricular Fibrillation in Patients With an Implantable Cardioverter-Defibrillator or Cardiac Resynchronization Therapy Defibrillator: A Multicenter Cohort Study
Source: Front Cardiovasc Med. 2020 Nov 30;7:610629. doi: 10.3389/fcvm.2020.610629 (PMC7734049; doi:10.3389/fcvm.2020.610629)
Supplement: Supplementary file 1 [file Data_Sheet_1.docx]

Supplementary Material

1. **Supplementary Text S1**

Programming settings were as follows: the basic pacing rate was 40-60 beats per minute (bpm), target VT monitor zone was 140-170 bpm, target VT therapy zone was over 170-210 bpm, and VF zone was over 210 bpm. In VT therapy zone, 2-3 bursts of anti-tachycardia pacing (ATP) were delivered, followed by high-energy shock for persisting episodes. In VF zone, high-energy shock alone was used. The detection interval was 26 beats in VT zone with a 20-beats redetection. And the detection interval was 12 out of 16 beats in VF zone. Other programmable parameters are determined by individual doctors. VT refers to spontaneous ventricular depolarization with a frequency of more than 100 bpm, for 3 or more consecutive times. The width of QRS is usually wider than 120ms. VF refers to the disordered agitation of the ventricle, which leads to the regular and orderly agitation and the disappearance of the systolic and diastolic function of the ventricle. Its electrocardiogram (ECG) is as follows: a constant shift in axis and morphology of the electrogram is accompanied by marked and variable changes in electrogram amplitude.

In our study, ICD/CRT-D was equipped with Biotronik SMART® algorithm which could automatically analyze the waveform and frequency of ECG to distinguish VT/VF and supraventricular tachycardia (including atrial fibrillation, atrial flutter, and sinus tachycardia). Additionally, the tachycardia events could be monitored by the ICD/CRT-D and automatically transmitted to the home monitoring system. Two cardiologists reviewed the intra-cardiac electrograms (IEGM) of tachycardia events in a blinded manner to further confirm the event as VT/VF or supraventricular tachycardia and analyzed the VAs to assess whether the patient received the appropriate ICD/CRT-D therapy. When there was a disagreement on the IEGM reading and ICD/CRT-D therapy, a third cardiologist was responsible for a conclusive opinion.

1. **Supplementary Table S1 Comparison of baseline characteristics of study population between complete data set and missing data set**

|  | **Complete data set**  **(n=970)** | **Missing data Set**  **(n=35)** | **P-value** |
| --- | --- | --- | --- |
| BMI | 23.4 ± 3.0 | 23.9 ± 4.9 | 0.364 |
| Age at implantation, years | 60.3 ± 13.5 | 63.8 ± 16.9 | 0.168 |
| SBP, mmHg | 124.5 ± 17.4 | 129.7 ± 18.4 | 0.100 |
| DBP, mmHg | 76.9 ± 10.9 | 80.5 ± 9.7 | 0.067 |
| Male | 707 (72.9%) | 25 (71.4%) | 0.849 |
| NYHA, Class III/IV | 484 (49.9%) | 15 (42.9%) | 0.124 |
| Primary prevention | 576 (59.4%) | 20 (57.1%) | 0.791 |
| CRT-D | 266 (27.4%) | 8 (22.9%) | 0.154 |
| Ischemic cardiomyopathy | 324 (33.4%) | 12 (34.3%) | 0.913 |
| Dilated cardiomyopathy | 238 (24.5%) | 7 (20.0%) | 0.539 |
| Hypertrophic cardiomyopathy | 37 (3.8%) | 2 (6.0%) | 0.899 |
| Long QT syndrome | 12 (1.2%) | 1(2.9%) | 0.943 |
| Hypertension | 305 (31.4%) | 10 (32.3%) | 0.719 |
| Diabetes mellitus | 101 (10.4%) | 1 (3.2%) | 0.146 |
| Stroke | 18 (1.9%) | 0 (0.0%) | 0.869 |
| Atrial fibrillation | 104 (10.7%) | 0 (0.0%) | 0.078 |
| Pre-implant syncope | 194 (20.0%) | 4 (11.4%) | 0.201 |
| LVEF, % | 42.5 ± 14.9 | 43.6 ± 14.2 (n=34) | 0.683 |
| LVEDD, mm | 58.8 ± 13.1 | NA |  |
| β-Blocker | 566 (58.4%) | 19 (54.3%) | 0.032 |
| Amiodarone | 290 (29.9%) | 16 (45.7%) | 0.046 |
| ACEI or ARB | 360 (37.1%) | 10 (28.6%) | 0.303 |
| Loop diuretic | 280 (28.9%) | 9 (25.7%) | 0.686 |
| Aldosterone antagonists | 363 (37.4%) | 11 (31.4%) | 0.471 |
| **Outcome**  VT/VF | 352 (36.3) | 11 (31.4%) | 0.556 |

Continuous data and categorical data were given as mean+SD and number (percentage), respectively.

**Abbreviations:** ACEI, angiotensin-converting enzyme inhibitor; ARB, angiotensin receptor blocker; BMI, body mass index; CRT-D, cardiac resynchronization therapy defibrillator; DBP, diastolic blood pressure; LVEF, left ventricular ejection fraction; LVEDD, left ventricular end-systolic dimension; NYHA, New York Heart Association; SBP, systolic blood pressure; VT/VF, ventricular tachycardia /ventricular fibrillation.

**3. Supplementary Table S2 Univariate Cox proportional hazards regression analysis of VT/VF**

|  | HR (95% CI) | P-value |
| --- | --- | --- |
| Male | 1.54 (1.19, 1.99) | 0.0009 |
| Age at implantation, years | 0.99 (0.98, 1.00) | 0.0217 |
| NYHA, Class III/IV | 1.09 (0.89, 1.35) | 0.3958 |
| SBP, mmHg | 1.00 (0.99, 1.01) | 0.9886 |
| DBP, mmHg | 1.01 (1.00, 1.02) | 0.2254 |
| Primary prevention | 0.91 (0.74, 1.12) | 0.3766 |
| CRT-D | 1.05 (0.83, 1.32) | 0.6946 |
| Ischemic cardiomyopathy | 1.03 (0.82, 1.28) | 0.7987 |
| Dilated cardiomyopathy | 1.17 (0.92, 1.49) | 0.1894 |
| Hypertrophic cardiomyopathy | 0.88 (0.50, 1.53) | 0.6405 |
| Long QT syndrome | 1.30 (0.58, 2.92) | 0.5234 |
| Hypertension | 0.93 (0.74, 1.17) | 0.535 |
| Diabetes mellitus | 1.13 (0.80, 1.59) | 0.4851 |
| Stroke | 0.88 (0.39, 1.98) | 0.761 |
| Atrial fibrillation | 1.56 (1.15, 2.11) | 0.0045 |
| Pre-implant syncope | 1.17 (0.91, 1.51) | 0.2097 |
| LVEF, % | 0.99 (0.98, 1.00) | 0.0121 |
| LVEDD, mm | 1.01 (1.01, 1.02) | 0.0011 |
| β-Blocker | 1.16 (0.93, 1.43) | 0.1872 |
| Amiodarone | 0.96 (0.77, 1.21) | 0.7595 |
| ACEI or ARB | 0.90 (0.73, 1.12) | 0.3634 |
| Loop diuretic | 0.92 (0.73, 1.17) | 0.5062 |
| Aldosterone antagonists | 1.06 (0.85, 1.31) | 0.624 |

Abbreviations are shown in Table 1
